# Insights into the Social Behavior of Surface and Cave-Dwelling Fish (*Poecilia mexicana*) in Light and Darkness through the Use of a Biomimetic Robot

**DOI:** 10.3389/frobt.2018.00003

**Published:** 2018-02-05

**Authors:** David Bierbach, Juliane Lukas, Anja Bergmann, Kristiane Elsner, Leander Höhne, Christiane Weber, Nils Weimar, Lenin Arias-Rodriguez, Hauke J. Mönck, Hai Nguyen, Pawel Romanczuk, Tim Landgraf, Jens Krause

**Affiliations:** ^1^Humboldt-Universität zu Berlin, bologna.lab, Q-Team Programm, Berlin, Germany; ^2^Department of Biology and Ecology of Fishes, Leibniz-Institute of Freshwater Ecology and Inland Fisheries, Berlin, Germany; ^3^Faculty of Life Sciences, Thaer Institute, Humboldt University of Berlin, Berlin, Germany; ^4^División Académica de Ciencias Biológicas, Universidad Juárez Autónoma de Tabasco (UJAT), Villahermosa, Tabasco, Mexico; ^5^Freie Universität Berlin, FB Mathematik u. Informatik, Berlin, Germany; ^6^Department of Biology, Institute for Theoretical Biology, Humboldt Universität zu Berlin, Berlin, Germany

**Keywords:** RoboFish, *Poecilia mexicana*, cave molly, Atlantic molly, biomimetic robot

## Abstract

Biomimetic robots (BRs) are becoming more common in behavioral research and, if they are accepted as conspecifics, allow for new forms of experimental manipulations of social interactions. Nevertheless, it is often not clear which cues emanating from a BR are actually used as communicative signals and how species or populations with different sensory makeups react to specific types of BRs. We herein present results from experiments using two populations of livebearing fishes that differ in their sensory capabilities. In the South of Mexico, surface-dwelling mollies (*Poecilia mexicana*) successfully invaded caves and adapted to dark conditions. While almost without pigment, these cave mollies possess smaller but still functional eyes. Although previous studies found cave mollies to show reduced shoaling preferences with conspecifics in light compared to surface mollies, it is assumed that they possess specialized adaptations to maintain some kind of sociality also in their dark habitats. By testing surface- and cave-dwelling mollies with RoboFish, a BR made for use in laboratory experiments with guppies and sticklebacks, we asked to what extent visual and non-visual cues play a role in their social behavior. Both cave- and surface-dwelling mollies followed the BR as well as a live companion when tested in light. However, when tested in darkness, only surface-dwelling fish were attracted by a live conspecific, whereas cave-dwelling fish were not. Neither cave- nor surface-dwelling mollies were attracted to RoboFish in darkness. This is the first study to use BRs for the investigation of social behavior in mollies and to compare responses to BRs both in light and darkness. As our RoboFish is accepted as conspecific by both used populations of the Atlantic molly only under light conditions but not in darkness, we argue that our replica is providing mostly visual cues.

## Introduction

Biomimetic robots (BRs) are becoming more common in behavioral research (Webb, 2000; Krause et al., 2011; Butail et al., 2015). One of the major advantages of BRs is that social interactions that are often characterized by mutual influences (Herbert-Read et al., 2012; Jolles et al., 2017) and feedbacks between multiple individuals (Harcourt et al., 2009) become in part controllable by the experimenter (Krause et al., 2011). Thus, standardized testing and new forms of experimental manipulations of social interactions can be achieved using BRs as interaction partners. However, investigations of social behavior become only meaningful when live animals accept BRs as conspecifics [(Landgraf et al., 2016), see similar views for computer animations in behavioral ecology (Chouinard-Thuly et al., 2017)]. At the moment, the number of animal species that respond to BRs as conspecifics is quite small. Thus, it is urgently needed to explore which cues emanating from BRs are crucial for the acceptance as a conspecific [reviewed in Landgraf et al. (2016)]. Tinbergen (1948) proposed that only a small subset of perceivable cues are actual signals (“social releasers”). They can be species specific, and animals often use sets of multiple cues to assess their (social) environment (Candolin, 2003). The identification of relevant cues and their realistic imitation is one of the most challenging parts in developing BRs (Krause et al., 2011). Since different species possess different sensory capabilities (Burnett, 2011), comparing the response of species with known differences in sensory ecologies toward BRs might help developers to understand the cues that are most important to establish social acceptance of their respective BRs. As an obvious by-product, BRs can help researchers to gain a much better understanding of social interactions in species, population, or ecotypes with different ecological and evolutionary backgrounds. In summary, both developers and biologists using BRs will benefit from a broader list of species investigated with BRs.

Here, we explored whether two populations of the Atlantic molly (*Poecilia mexicana*, a cave-dwelling and a surface-dwelling ecotype) accept a BR as a conspecific that was initially developed to meet the requirements of the closely related guppy (*Poecilia reticulata*), a “model organism” in many different biological fields (Magurran, 2005). Both populations of cave- and surface-dwelling mollies differ in their evolutionary background and consequently also in their assumed sensory capabilities and social tendencies. Although cave mollies still possess functional albeit smaller eyes than their surface-dwelling counterparts (Körner et al., 2006; Eifert et al., 2014), their non-visual systems seem to be much better developed (Parzefall, 1969, 1970, 2001; Peters et al., 1973; Parzefall et al., 2007) with recent investigations pointing out that both chemical and mechanosensory communication is more pronounced in cave mollies compared to surface dwellers (Rüschenbaum and Schlupp, 2013; Jourdan et al., 2016). Furthermore, cave mollies were previously found to be less attracted to conspecifics in dichotomous choice tests under normal light conditions and hence are assumed to be less social than the closely related surface ecotypes (Plath and Schlupp, 2008).

We tested the social behavior of surface- and cave-dwelling Atlantic mollies with both a live conspecific and a BR under both light and dark conditions. We hypothesized that cave mollies should be generally less social toward (e.g., do not follow closely) live conspecifics and BRs compared to surface-dwelling mollies in light but should be better able to maintain some sociality in darkness with both a live conspecific and a BR compared to surface-dwelling fish. Our study not only tests for differences in social behavior of surface and cave-dwelling fish but also tests whether BRs constructed as mobile replicas are accepted as conspecifics when visual cues are omitted (as in darkness).

## Materials and Methods

### Test Fish and Their Maintenance

**Figure 1:**
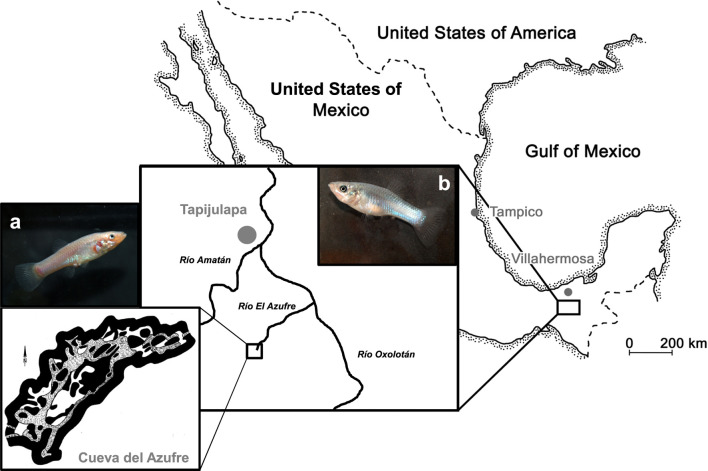
Map of our study system. Both tested molly populations originate from the South of Mexico near the city of Tapijulapa, federal state of Tabasco. Here, ancestral forms of *Poecilia mexicana* colonized both surface (b, surface-dwelling molly) as well as cave (a, cave molly) habitats.

In this study, we used second-generation lab-reared descendants of wild-caught fish from two populations of the Atlantic molly (*P. mexicana*) that were caught during field trips to the Tacotalpa river system in Tabasco, Mexico (Tobler et al., 2011; Plath et al., 2013). Our surface population originated from the Río Oxolotán, a tributary to the Río Grijalva, while our cave population stemmed from chamber 7 of the cave Cueva del Azufres (Figure 1). Fish were reared in randomly outbred mixed-sex stocks at the Laboratory of Genetics and Ecophysiology from the Academic Division for Biological Sciences-UJAT. Some of them were transported to the Department of Biology and Ecology of Fishes at the Thaer-Institute for Life Sciences at Humboldt University of Berlin for the present experiment. For the rearing prior to any experiment, we used a light regime of 12 h light:12 h darkness that resembles the natural surface habitats and maintained water temperature at 26°C. Prior to experiments, test fish were taken from their stock tanks (80-L) and transferred into 54-L tanks in groups of 20 individuals with equal sex ratio. Those 54-L tanks were covered with black plastic foil and could be run either with a 12:12 L:D light regime (6,000 vs. 0 lux) or in total darkness (0 lux; in case fish were later tested in darkness, see below). Fish were fed twice daily *ad libitum* with TetraMin flake food and live Chironomid larvae. Please note that all our test subjects have been raised under normal 12 h light:12 h dark conditions in the lab, and cave mollies thus might not show exactly the same behaviors compared to their wild counterparts that spend their whole lives in darkness. This was necessary to facilitate maintenance work and to ensure that all fish tested in darkness experienced the same treatment since it is impossible to raise surface mollies in darkness without high mortality rates (Riesch et al., 2011). Nevertheless, we acclimated those fish tested in darkness (surface and cave mollies) to complete dark conditions for 1 week prior to our tests. Such an approach follows previous protocols for that species (Plath et al., 2003, 2004).

### The BR: RoboFish

**Figure 2:**
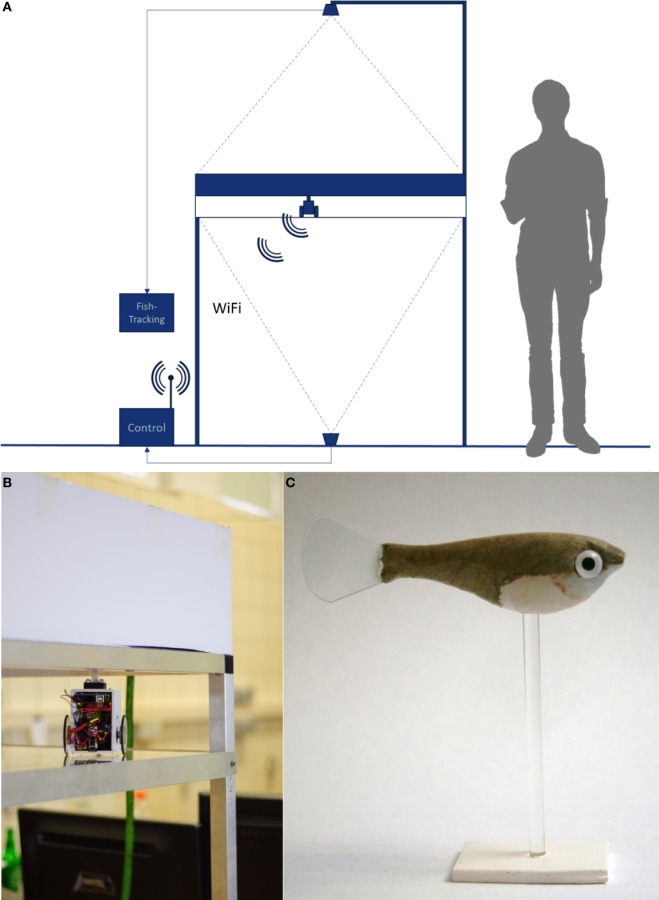
The RoboFish system. **(A)** Experimental setup showing the test tank and bottom as well as top view cameras. The robot is running on a transparent second level below the test tank and is connected via Wi-Fi to the controlling computers. **(B)** Robot close-up below the test tank. **(C)** A molly like replica equipped with glass eyes.

**Figure 3:**
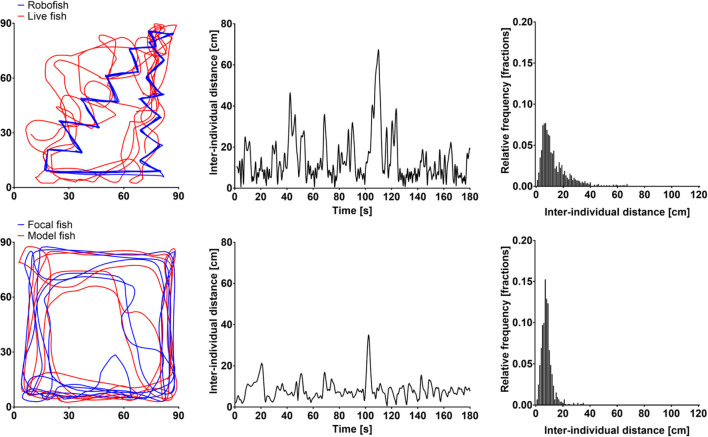
Example tracks of a surface-dwelling molly with RoboFish (top) and a live companion (bottom). The swam trajectories (left), the interindividual distance during the 180-s observation period (center) as well as its distribution (right) are shown.

Our RoboFish system consists of a glass tank (88 cm × 88 cm) that is filled to a level of 15 cm with aged tap water. The tank is placed on an aluminum rack at about 1.40 m above ground (Figure 2A). The two-wheeled differential drive robot moves below the tank on a transparent platform (Figure 2B). It carries a neodymium magnet directed to the bottom side of the tank. A three-dimensional (3D)-printed fish replica (Figure 2C) is attached to a magnetic base, which aligns with the robot. Hence, the replica can be moved directly by the robot (Figure 2B). On the ground, a camera is facing upward to track the robot. A second camera (IR-sensitive Bosch Dinion 1080p) is fixed above the tank to track both live fish and replica. The entire system is enclosed in a black, opaque canvas to minimize exposure to external disturbances. For trials in light, the tank was illuminated from above with artificial LED lights reproducing the daylight spectrum (2,000 lux). For trials in darkness, we used four IR spots to light the tank, which cannot be perceived by the fish (Körner et al., 2006) but allows our above-tank camera to record. Two personal computers are used for system operation: one PC tracks (bottom camera) and steers the robot *via* Wi-Fi, whereas a second PC records the video feed of the top camera. The RoboFish moves on a predefined trajectory through the test tank (also called “open-loop” steering). The trajectory used in all described experiments is given in Figure 3; RoboFish swims on a continuous zigzag path through the tank. For more detailed information on RoboFish operation modes and construction, see the study by Landgraf et al. (2016).

### Experimental Setup: Social Interactions under Two Different Light Conditions

To investigate how surface- and cave-dwelling populations of the Atlantic molly differ in their social behavior in both light and dark conditions, we observed the interactions of live fish with either RoboFish (*n* = 3 live fish tested for each population and light regime) or with another live conspecific (*n* = 3 pairs of live fish for each population and light regime). The 3D-printed fish replica was modified to match the appearance of *P. mexicana* (Figure 2C). The size of the replica (SL: 35 mm) was derived from the mean standard length of all test fish (ranging between 28.77 and 49.03 mm). The replica was situated 0.5 cm below the water surface in accordance with the close-to-surface swimming behavior observed for both populations in the wild (Jourdan et al., 2014). We programmed RoboFish to an average speed of 10 cm/s (maximum speed of 27 cm/s). This is comparable to average speeds obtained for live fish in pilot experiments. The RoboFish swimming sequence was initiated immediately upon transferring the fish into the arena. We started to score social interactions for 3 min after both subjects were first within a range of four body lengths, a distance often assumed to indicate social interactions in poeciliid and other fishes (Croft et al., 2008). Similarly, during trials with conspecifics, two fish were transferred into the arena simultaneously and scoring for 3 min started when fish were moving and after being within a range of 12 cm (ca. 4 body lengths). The fish’s movements were tracked using EthoVision™ XT10.1 software (Noldus Information Technology), and the obtained XY position data were analyzed using customized Python scripts (Python Software Foundation).

### Statistical Analysis

Our first aim is to establish whether our focal live fish were socially attracted to their respective companions (either another live fish or RoboFish) in light and darkness. To do so, we compared average median distances between both subjects (“interindividual distance”; see Figure 3) in our trials to average median distances obtained for simulated random tracks (“null models”). To obtain “null models”, we randomly shuffled focal fish’s XY positions at each sampled time step (e.g., by randomly changing order of time steps) and afterward calculated distance between focal fish’s XY position to that of the companion’s XY position for all time steps. Doing so kept all focal fish’s positions but links them randomly with those of the companion. Average medians of interindividual distances of real and simulated tracks were then compared *via* Wilcoxon’s rank test (one-tailed, null models are assumed to have greater median interindividual distances), separated by species, companion, and light treatment.

Our second aim is to establish whether surface- and cave-dwelling Atlantic mollies differ in their social behavior (e.g., their shoaling tendency in pairs of live fish or their following tendencies toward RoboFish) and whether light conditions differentially affect social behavior of surface- and cave-dwelling fish. Therefore, we compared average medians of interindividual distances between surface- and cave-dwellers using Mann–Whitney *U*-tests, separated by light treatment (in light or darkness) and social partner treatment (live companion or RoboFish). Please note that our sample sizes are quite small (*N* = 3 per treatment), which is due to the intense tracking efforts under dark conditions and limited numbers of fish available. Thus, non-significant differences can be a result of low statistical power (in our case beta errors of non-significant tests ranged between 0.05 and 0.40). However, in case of non-significant differences, values were always overlapping.

## Results

**Figure 4:**
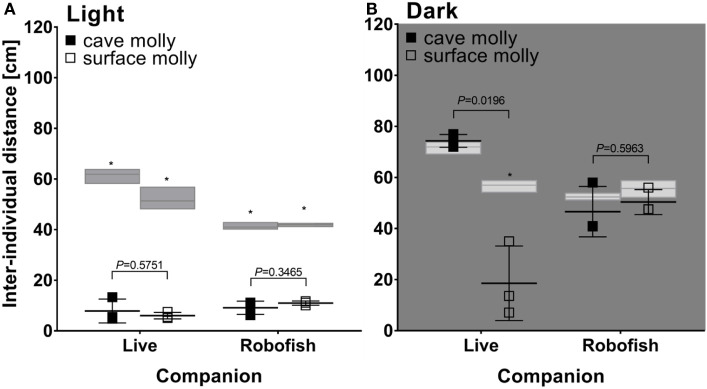
Social behavior of surface and cave-dwelling mollies tested with live conspecifics and RoboFish in light **(A)** and darkness **(B)**. Shown are median interindividual distances along with the results of *U*-tests (*P* values above bars) comparing cave- and surface-dwelling mollies in each treatment. Gray bars represent median and range of simulated interindividual distances for each treatment, and asterisks indicate a significant difference between simulated and real data in Wilcoxon’s rank tests (*P* < 0.05).

In light, both cave- and surface-dwelling mollies were similarly strongly attracted to live companions and RoboFish. This was evidenced by significantly smaller interindividual distances among subjects in real interactions compared to simulated tracks (e.g., rank tests comparing median interindividual distances in real interactions and simulated “null models” were significant; see Figure 4A). Also, there was no significant difference detectable between the interindividual distances obtained from cave- or surface-dwelling fish when tested with a live companion or RoboFish (*U*-tests non-significant; see Figure 4A). We provide example tracks and interindividual distance plots for RoboFish and live–live interactions of a surface molly in light in Figure 3.

In darkness, cave mollies were not attracted to either live companions or RoboFish. Consequently, we found no significant difference between real and simulated tracks (rank tests not significant; Figure 4B). Interestingly, surface mollies still showed a strong social attraction toward live companions in darkness with significantly smaller interindividual distances compared to simulated random tracks (Figure 4B). However, as seen in cave mollies, surface mollies were not attracted to RoboFish (rank test not significant; Figure 4B). Thus, despite our low overall sample size, we found significant differences between cave- and surface-dwelling fish in regard to their social behavior with a live companion in darkness (significance *U*-test; Figure 4B) but not Robofish (non-significance *U*-test; Figure 4B).

## Discussion

In light, we found both surface- and cave-dwelling mollies to be similarly strongly attracted by a live conspecific, which contradicts the previously proposed reduced sociality of cave mollies (Plath and Schlupp, 2008). As found in tests with live conspecifics, both ecotypes were following closely a moving BR—RoboFish. This shows the utility of BRs for the study of collective behavior especially in poeciliid fishes (Polverino et al., 2013; Landgraf et al., 2016). However, when tested in darkness, both ecotypes did not follow RoboFish, suggesting that our BR was providing sufficient social cues only when visual inspection was possible. Hence, robotically driven replicas as used in our experiments seem to exploit exclusively visual communication channels. Interestingly, cave fish were also no longer attracted by a live conspecific when tested in darkness, whereas surface-dwellers still showed a significant attraction toward live conspecifics. This contrasts our initial prediction that predominately cave fish with their increased non-visual sensing (Parzefall, 2001) should be able to maintain some degree of sociality also in the dark.

Plath and Schlupp (2008) found that cave mollies from two independently colonized caves (including the population from the Cueva del Azufre also used in our experiments) showed reduced shoaling tendencies when either only visual (stimulus group was presented behind a glass barrier) or both visual and non-visual communications (group presented behind a mesh-wired barrier) was allowed. Thus, the authors concluded that “observed reduction in shoaling in the two cave populations represents a parallel evolutionary process” (Plath and Schlupp, 2008). So, why are cave mollies similarly attracted by live conspecifics and RoboFish compared to surface-dwelling mollies when tested in light in our full-contact experiments? The assumed low sociality of cave mollies was based on dichotomous choice tests in light in which cave- and surface-dwellers had to choose among a group of conspecifics or an empty compartment in the test aquarium (Plath and Schlupp, 2008). While this is a classic and commonly used method to establish shoaling tendencies in small fish (Wright and Krause, 2006), we argue that full contact designs as in our study might lead to different results (Ziege et al., 2012). In addition, technological advances make it easier for the experimenter and thus more common to track animals’ movements while they interact unconstrained (Herbert-Read et al., 2011, 2012; Katz et al., 2011; Jolles et al., 2017). Future studies should then focus on comparative approaches evaluating strengths and short comings of either method.

While our tests in light provided cave and surface fish with both visual and non-visual cues and each ecotype might have predominately used one or the other to associate with a live or artificial companion, our tests in darkness omitted visual communication. In experiments using mesh-wired barriers in dichotomous choice tests under dark conditions, Plath et al. (2004, 2005) found only cave mollies to be able to exercise mate choice, an ability that was also confirmed in the wild (Bierbach et al., 2013a). This was attributed to cave mollies exhibiting evolutionary acquired enhanced lateral line (Parzefall, 2001; Parzefall et al., 2007) as well as chemical sensing of conspecifics (Rüschenbaum and Schlupp, 2013; Jourdan et al., 2016). Thus, we initially hypothesized that cave mollies, although assumed to have an inherent weaker social tendency, should show stronger social attraction in darkness compared to surface fish. We found the opposite with cave mollies showing no social attraction but surface-dwellers were still significantly attracted to a live companion. Shoaling is a behavioral adaptation to predation risk (Krause and Ruxton, 2002), which is strongly reduced in the cave habitat. The Cueva del Azufre is free of piscivorous fish as well as birds, and the only predators preying upon cave mollies are giant water bugs of the genera *Belostoma* (Tobler et al., 2007) as well as pisaurid and theraphosid spiders (Horstkotte et al., 2010) and freshwater crabs (Klaus and Plath, 2011). All these species are sit-and-wait predators that prey from the pool edges and thus have only very limited attack ranges. Thus, it is likely that shoaling does not provide mollies with antipredator benefits in the cave, and there is no evolutionary pressure to maintain shoaling behavior by cave mollies in darkness. This view is supported by experiments showing that cave mollies exhibit reduced avoidance when confronted with fish predators (Bierbach et al., 2013b). It is also possible that cave mollies context dependently adjust their shoaling tendencies in darkness but not in light. This seems to be a unique feature of cave fish as surface-dwelling fish, also habituated to darkness for 1 week, still showed significant shoaling tendencies, probably by using non-visual communication channels like lateral line sensing and conspecific chemical cues (see above). As surface fish might experience predation also in darkness (e.g., during night), maintaining shoaling under dark conditions can be still beneficial. As our sample size was small (see methods) and thus statistical evaluation limited, we recommend future studies to focus on shoaling differences of surface- and cave-dwelling mollies using up-to-date full contact designs and position tracking approaches.

As both cave- and surface-dwelling mollies did not show any social attraction toward RoboFish in darkness, we conclude that our replica is providing only sufficient visual cues but lack other non-visual ones that are important to be recognized as a conspecific in darkness. It is known that tail beating of fish replicas can enhance acceptance probably by stimulating the lateral line system (Marras and Porfiri, 2012), and it seems that a pure swimming (even with direction changes as in our zig-zagged trajectories) does not provide enough similar stimulation. In non-visually communicating animals like weak-electric fishes or insects, researchers tried to mimic species-specific cues by either rebuilding electric discharges at the replica (Donati et al., 2016) or by applying conspecific odors to the replica (Halloy et al., 2007; Landgraf et al., 2011). Furthermore, some researchers now focus on the development of replicas that provide multiple cues (Shi et al., 2013; Phamduy et al., 2014; Donati et al., 2016; Romano et al., 2017). Future research might focus on exploring which non-visual cues are important for poecillid fishes by step-wise equipping replicas with different artificial cues and comparing the response of live fish in light and darkness. In addition, a comparison with other cave fish will be fruitful as well since several cave ecotypes are blind and thus exclusively rely on non-visual cues (Jeffery et al., 2003). Overall, RoboFish (and similar biomimetic systems) can be a strong tool to investigate social behavior of fish in a standardized way.

## Ethical Statement

Fish brood stocks were collected under the authorization of the Mexican government (DGOPA.09004.041111.3088, PRMN/DGOPA-003/2014, PRMN/DGOPA-009/2015, and PRMN/DGOPA-012/2017, issued by SAGARPA-CONAPESCA-DGOPA). Experiments reported in this study were carried out in accordance with the recommendations of “Guidelines for the treatment of animals in behavioral research and teaching” (published in Animal Behavior 1997). The protocol was approved by the LaGeSo Berlin under the registration number 0117/16.

## Author Contributions

DB, JL, AB, KE, LH, CW, NW, TL, PR, and JK designed the study. DB, JL, LA-R, and JK caught the fish. DB, HM, HN, and TL built the robot system. DB, JL, AB, KE, LH, CW, NW, and HN performed the experiments, DB, KE CW, and PR tracked the videos. DB analyzed the data. All authors interpreted the data and approved the submitted manuscript version.

## Conflict of Interest Statement

The authors declare that the research was conducted in the absence of any commercial or financial relationships that could be construed as a potential conflict of interest.
